# How Does Deep Neural Network-Based Noise Reduction in Hearing Aids Impact Cochlear Implant Candidacy?

**DOI:** 10.3390/audiolres14060092

**Published:** 2024-12-13

**Authors:** Aniket A. Saoji, Bilal A. Sheikh, Natasha J. Bertsch, Kayla R. Goulson, Madison K. Graham, Elizabeth A. McDonald, Abigail E. Bross, Jonathan M. Vaisberg, Volker Kühnel, Solveig C. Voss, Jinyu Qian, Cynthia H. Hogan, Melissa D. DeJong

**Affiliations:** 1 Division of Audiology, Department of Otolaryngology-Head and Neck Surgery, Mayo Clinic, Rochester, MN 55902, USA; 2 Sonova Canada Inc., Kitchener, ON N2E 1Y6, Canadasolveigchristina.voss@sonova.com (S.C.V.);; 3 Sonova AG, 8712 Stäfa, Switzerland

**Keywords:** cochlear implants, hearing aids, deep neural network

## Abstract

Background/Objectives: Adult hearing-impaired patients qualifying for cochlear implants typically exhibit less than 60% sentence recognition under the best hearing aid conditions, either in quiet or noisy environments, with speech and noise presented through a single speaker. This study examines the influence of deep neural network-based (DNN-based) noise reduction on cochlear implant evaluation. Methods: Speech perception was assessed using AzBio sentences in both quiet and noisy conditions (multi-talker babble) at 5 and 10 dB signal-to-noise ratios (SNRs) through one loudspeaker. Sentence recognition scores were measured for 10 hearing-impaired patients using three hearing aid programs: calm situation, speech in noise, and spheric speech in loud noise (DNN-based noise reduction). Speech perception results were compared to bench analyses comprising the phase inversion technique, employed to predict SNR improvement, and the Hearing-Aid Speech Perception Index (HASPI v2), utilized to predict speech intelligibility. Results: The spheric speech in loud noise program improved speech perception by 20 to 32% points as compared to the calm situation program. Thus, DNN-based noise reduction can improve speech perception in noisy environments, potentially reducing the need for cochlear implants in some cases. The phase inversion method showed a 4–5 dB SNR improvement for the DNN-based noise reduction program compared to the other two programs. HASPI v2 predicted slightly better speech intelligibility than was measured in this study. Conclusions: DNN-based noise reduction might make it difficult for some patients with significant residual hearing to qualify for cochlear implantation, potentially delaying its adoption or eliminating the need for it entirely.

## 1. Introduction

Cochlear implants (CIs) are highly effective medical devices for restoring hearing in patients with varying degrees of hearing loss. Despite their effectiveness, there is considerable international variation in the specific audiometric and speech perception criteria used to determine eligibility for cochlear implantation. Vickers et al. and Van de Heyning et al. conducted independent studies to assess international variations in cochlear implant candidacy criteria. Both studies involved 17 different countries in their analysis [1,2]. A key finding from both studies was the widespread use of audiometric thresholds as a primary criterion for cochlear implant candidacy. Specifically, an average hearing loss of 75–80 dB HL at frequencies above 1 kHz is commonly accepted. However, the studies also revealed differences in the use of speech perception tests. While Vickers et al. reported that seven countries rely on word and/or sentence recognition scores in both quiet and noisy conditions, Van de Heyning et al. primarily found the use of word recognition scores. The latter study also noted a range of 40–60% word recognition scores in quiet conditions for unaided hearing and 60–100% for aided hearing. In the United States, the Centers for Medicare & Medicaid Services (CMS) and private insurers provide guidelines for CI eligibility based on speech perception criteria. CMS requires patients to have bilateral moderate-to-profound hearing loss with speech perception scores below 60% in their best aided condition, as measured by sentence testing [3]. However, these guidelines lack specificity regarding speech presentation level, background noise, and sentence material, which can all impact scores.

Many clinics in the United States assess CI candidacy using AzBio sentences [4] in both quiet and noisy listening conditions [5,6,7,8]. The sentences and noise are typically presented from a single loudspeaker positioned in front of the speaker, with signal-to-noise ratio (SNR) of 10 or 5 dB often used to qualify patients for cochlear implantation. Individuals qualified at this SNR level often demonstrate significant listening improvements in both quiet and noisy settings following implantation [8]. This assessment approach aims to expand candidacy to include patients with significant residual hearing and poor speech comprehension.

Modern hearing aids employ noise reduction signal processing and directional microphones to reduce background noise and enhance speech perception. Traditional noise reduction signal processing is most beneficial for steady-state noises by predicting the noise envelope and attenuating the noise. However, benefits are further limited in less-predictable, fluctuating noises like multi-talker babble [9,10]. Directional microphones are effective when speech and noise sources are spatially separated [11]. However, directional microphones are less effective in scenes with high reverberation and if the target is further away. Also, due to the single-speaker test condition used in CI candidacy assessments in which speech and multi-talker babble are co-located, noise reduction signal processing and directional microphones are unlikely to significantly influence CI evaluation outcomes. This study does not account for the proven benefits of directional microphones but demonstrates that deep neural network technology can provide additional improvements in SNRs and even speech intelligibility, particularly when no beamformer benefit is anticipated.

With the advancement of artificial intelligence (hereinafter referred to as deep neural network [DNN]), modern hearing aids are now capable of using DNN-based sound cleaning [12]. DNN-based features are likely to provide significant and measurable improvements in speech perception in fluctuating noise, such as multi-talker babble, even when speech and noise are co-located, as is commonly the case in CI evaluations.

Conventional noise reduction algorithms improve speech by reducing background noise, usually by lowering gain during speech pauses to enhance the signal-to-noise ratio. While effective in some situations, these methods may not perform well in complex or rapidly changing sound environments. DNN-based noise reduction operates on a similar principle, identifying and removing unwanted noise while preserving speech. However, it uses a network with 4.5 million parameters, trained on 22 million sound samples, to improve performance across a wider range of conditions. The DNN processes a full-spectrum audio signal, divided into 64 frequency bands, each containing both amplitude and phase components. This comprehensive approach allows the DNN to more accurately separate speech from noise compared to conventional algorithms, which typically operate at lower frequency resolutions and use real-valued filter weights. By using a complex-valued filter, the DNN can more precisely reconstruct the speech signal. This approach contrasts with traditional methods, which primarily reduce gain in quieter segments, sometimes leading to a loss in audio quality. The DNN, by evaluating the entire signal, provides a more effective solution for reducing noise while maintaining speech intelligibility [13].

Raufer et al. found that compared to an omnidirectional reference, directional microphones improved the SNR by approximately 3.5 dB, single-channel noise cancellers improved the SNR by 2.3 dB, and the DNN-based noise reduction algorithm resulted in an SNR improvement of 5 dB. The improvement from directional microphones can be combined with the gains from the two noise reduction algorithms. Specifically, when combined with single-channel noise cancellers, the total SNR improvement reached was 5.8 dB. When paired with DNN-based noise reduction, the SNR improvement was even greater, reaching 8.5 dB [14].

In this study, we evaluated the impact of DNN-based noise reduction program on CI evaluation outcomes in patients with significant hearing loss. To assess the impact, Phonak Audéo Sphere Infinio 90 receiver-in-canal hearing aids were fitted with power domes and programmed with three manual programs: (1) a program optimized for calm listening environments, (2) a program designed for speech understanding in noisy situations, and (3) a program specifically tailored for spheric speech in loud noise. Sentence recognition tests were conducted in quiet and in the presence of multi-talker babble to quantify the performance differences between the three programs. To assess SNR improvements from DNN-based noise reduction, the phase inversion technique developed by Hagerman and Olofsson was employed [15]. While improvements in the SNR can make speech easier to understand, this relationship is not necessarily direct and does not always predict improvement in speech intelligibility. Therefore, to complement the speech intelligibility data gathered from participants, a model-based prediction of speech intelligibility was incorporated. For predicting speech intelligibility, the Hearing-Aid Speech Perception Index (HASPI) was utilized to determine whether modeling can accurately predict behavioral outcomes observed in participants [16,17,18].

## 2. Methods

### 2.1. Patient Evaluations

This study presents speech perception scores for ten patients with significant hearing loss. Five of these patients had bilateral hearing loss and were referred to our clinic for CI evaluation. Five other patients had bilateral hearing loss with a CI in one ear (see Table 1). These patients were evaluated without their CI but with a hearing aid in the contralateral ear, while the bilateral hearing-impaired patients referred for CI evaluation were fitted bilaterally. All participants underwent speech perception testing in both quiet and multi-talker babble conditions. These measurements were collected during their clinical visits to determine their eligibility for cochlear implantation. The retrospective review of test results from patient medical records was approved by the Mayo Clinic Institutional Review Board (24-010370).

Pure tone air- and bone-conduction audiometry was performed using an Otometrics Madsen Astera2 audiometer, ER-3A insert earphones, and B-71 bone vibrator. The audiometric thresholds were used to program a pair of Phonak Audéo Sphere Infinio 90 receiver in the canal hearing aids with power domes attached. All hearing aids were fit by matching the hearing aid output to the target gain recommended by DSL-v5 for adults [19].

Speech perception was evaluated using AzBio sentences presented at 60 dB SPL (fixed presentation level) in both quiet and multi-talker babble conditions at the SNRs of 5 and 10 dB. The long-term root mean square (RMS) level of the multi-talker babble noise was scaled to achieve 5 and 10 dB SNRs. Sentence testing was performed using a single loudspeaker located in front of the patient. Multi-talker babble was presented continuously while measuring sentence recognition. One list of 20 sentences was used to measure speech perception for each listening condition.

Sentence recognition scores were measured for three manual hearing aid programs, namely (1) calm situation, (2) speech in noise, and (3) spheric speech in loud noise, each linked to the associated settings of the hearing aid operating system, AutoSense OS. The calm situation program is the most sensitive to sound all around the hearing aid wearer. The speech in noise program utilizes a noise reduction signal processing algorithm and adaptive directional microphone technology. The spheric speech in loud noise program incorporates DNN-based noise reduction and adaptive directional microphone technology.

The “Speech in Noise” and “Spheric Speech in Loud Noise” programs in AutoSense OS are activated based on a sound scene classification criterion, which considers both the type of sound environment, and the environmental sound pressure level monitored by the hearing devices. The “Speech in Noise” program is triggered at approximately 60 dB SPL, while the “Spheric Speech in Loud Noise” program activates at levels above 70 dB SPL. These activation thresholds are not absolute and may vary depending on the specific characteristics of the background noise, but the stated values are generally applicable to typical acoustic environments.

#### Statistical Analysis

Speech perception scores were analyzed using SigmaPlot (version 15.4; Systat Software, Inc., San Jose, CA, USA). The Shapiro–Wilk test was employed to assess the normality of the data. A one-way analysis of variance (ANOVA) was conducted to determine significant differences in speech perception scores among the three manual hearing programs at SNRs of 10 and 5 dB. Post hoc pairwise comparisons using a Bonferroni correction were performed to identify specific differences between the programs. Statistical significance was set at a *p*-value of less than 0.05.

### 2.2. Hearing Aid Recordings and Experimental Setup

Hearing aid recordings were conducted in a sound-treated, semi-anechoic laboratory using a KEMAR manikin equipped with average adult pinna replicas and G.R.A.S. RA0045 ear simulators (GRAS Sound and Vibration, Lynge, Denmark). A Genelec 8331A speaker (Genelac, Iisalmi, Finland) was positioned one meter from the KEMAR to mimic the experimental setup used for the behavioral component of the study.

The electrical signal from a G.R.A.S. power module 12AK was captured using an RME Fireface 800 (Remote Media Engineering (RME), Haiming, Germany) soundcard interfaced with an Optiplex 7000 PC (Dell Technologies, Round Rock, TX, USA). Adobe Audition CC was used to record the input signal. The entire setup was calibrated using a G.R.A.S. 42AA pistonphone (GRAS Sound and Vibration, Lynge, Denmark) and an NTi-XL2 sound level meter (NTi Audio, Schaan, Liechtenstein).

Measurements were performed using the same speech and noise materials employed in the behavioral component of the study. Phonak Audéo Sphere Infinio 90 hearing aids, fitted with titanium custom slim tips (no venting), were programmed for either moderate or severe hearing loss (N4 or N5 hearing thresholds, respectively) [20] and fitted to DSL-v5 adult gain targets.

A total of 18 conditions were recorded, combining two standard audiogram configurations (N4, N5), three program settings (calm situation, speech in noise, spheric speech in loud noise), and three SNR levels of 0, 5, and 10 dB.

SNR analysis was conducted using the phase inversion technique [15]. This technique involves presenting two versions of a speech and noise sample: (1) the speech and noise are presented in their original phase and (2) the phase of the noise signal is inverted. The KEMAR outputs from these three versions of the signal are used to calculate the level of both speech and noise, from which the SNR associated with each hearing aid program can be determined.

For predicting speech perception and intelligibility, the recorded signals were analyzed using HASPI v2, which simulates the human auditory system to predict speech perception and intelligibility. HASPI version 2 (v2) incorporates an advanced auditory framework that simulates the effects of hearing loss, including reduced audibility, diminished non-linear compression, widened cochlear filtering, and inner hair cell synapse modeling [16,17,18]. This framework enables the prediction of speech intelligibility for a given speech signal. HASPI v2 analyzes the recording as follows. (1) Input: The tool requires the recorded test signal; a reference signal consisting of the clean; unprocessed sentences; their sampling rates; the listener’s hearing thresholds at 0.25, 0.5, 1, 2, 4, and 6 k Hz; and the reference sound pressure level (SPL) corresponding to a root mean square (RMS) value of 1. (2) Processing: Both the recorded and reference signals are processed through the simulated auditory system. (3) Output: The tool compares the outputs of the simulated system for the recorded and reference signals to predict speech perception and intelligibility.

## 3. Results

### 3.1. Patient Evaluation

**Figure 1:**
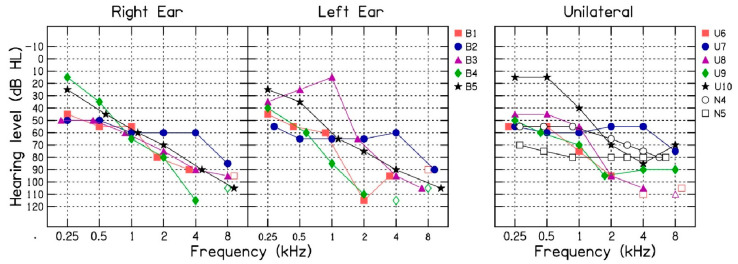
Air-conduction thresholds for five patients with bilateral (B1 to B5) and five patients with unilateral (U6 to U10) hearing loss and the N4 and N5 audiograms from Bisgaard et al. [16].

Figure 1 shows the left and right audiometric air-conduction thresholds for the five patients with bilateral (B1 to B5) hearing loss and five patients with unilateral (U6 to U10) hearing loss along with air-conduction thresholds for the N4 and N5 audiogram from Bisgaard et al. [20]. All patients had a moderate-to-profound degree of hearing loss. These audiometric thresholds were used to fit the new hearing aid either unilaterally or bilaterally.

**Figure 2:**
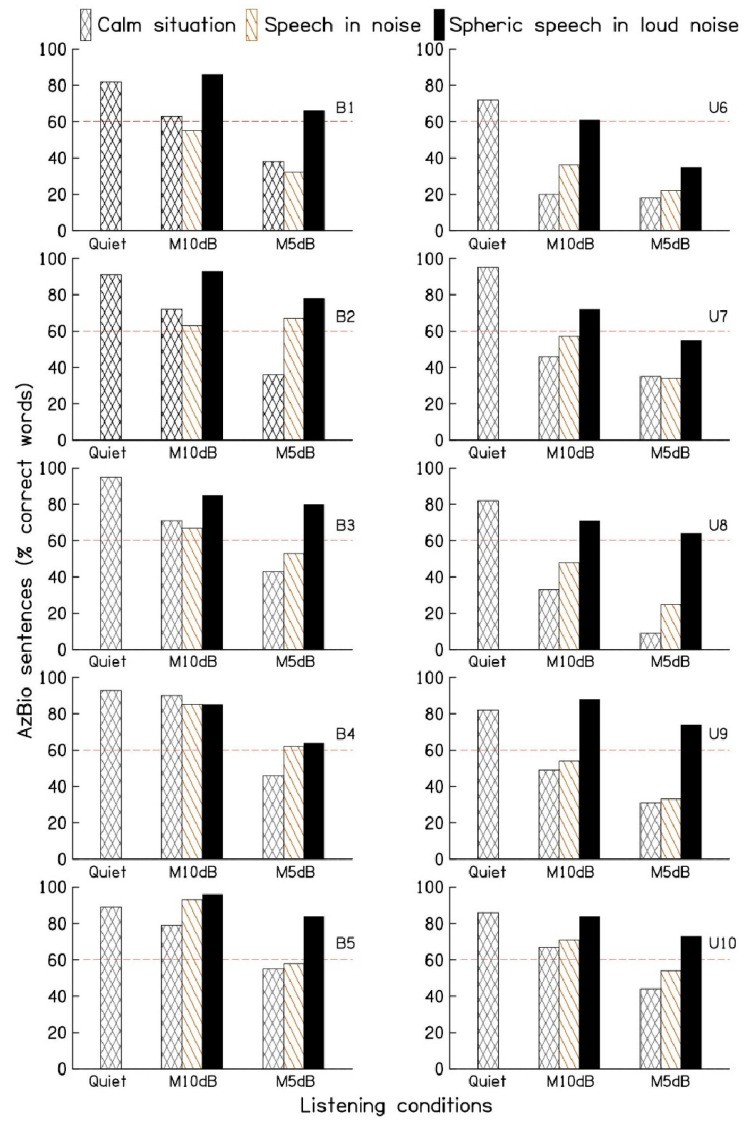
Sentence recognition scores in quiet and in the presence of multi-talker babble with a signal-to-noise ratio of 10 and 5dB for three different manual programs: calm situation, speech in noise, and spheric speech in loud noise. The 60% criterion line (red dashed line) signifies the threshold below which a participant may be considered a candidate for cochlear implantation.

Figure 2 depicts the speech perception scores of the ten participants for the three different hearing aid programs, namely calm situation, speech in noise, and spheric speech in loud noise. The dashed line in Figure 2 indicates the 60% criterion, below which participants may be eligible for cochlear implantation. On average, participants achieved an 87% ± 2.3% (standard error) sentence recognition score in quiet conditions. When exposed to multi-talker babble at the 10 dB SNR, scores decreased to 59% ± 6.8% for the calm situation program, 63% ± 5.3% for the speech in noise program, and 82% ± 3.4% for the spheric speech in loud noise program. With a 5 dB SNR, scores dropped further to 36% ± 4.2%, 44% ± 5.2%, and 67% ± 4.5%, respectively.

Compared to the calm situation program, the spheric speech in loud noise program showed average improvements of 23% points and 32% points at the 10 and 5 dB SNRs, respectively. Similarly, the spheric speech in loud noise program showed average improvements of 20% points and 23% points at the 10 and 5 dB SNRs, respectively, compared to the speech in noise program.

The Shapiro–Wilk test confirmed the normal distribution of the data. A one-way analysis of variance (ANOVA) was conducted to compare speech perception scores across the three programs for the ten participants, with the expectation that intelligibility would be highest in the spheric speech in loud noise program. ANOVA revealed statistically significant differences in sentence recognition scores among the programs under both 10 dB (F (2, 10) = 4.604, *p* < 0.05) and 5 dB (F (2, 9) = 12.104, *p* < 0.001) multi-talker babble conditions.

Pairwise comparisons using the Bonferroni *t*-test indicated significant differences (*p* < 0.05) between the spheric speech in loud noise program and the calm situation and speech in noise programs for both noise levels. However, no significant differences were found between the calm situation and speech in noise programs under either multi-talker babble conditions.

### 3.2. Bench Evaluation

**Figure 3:**
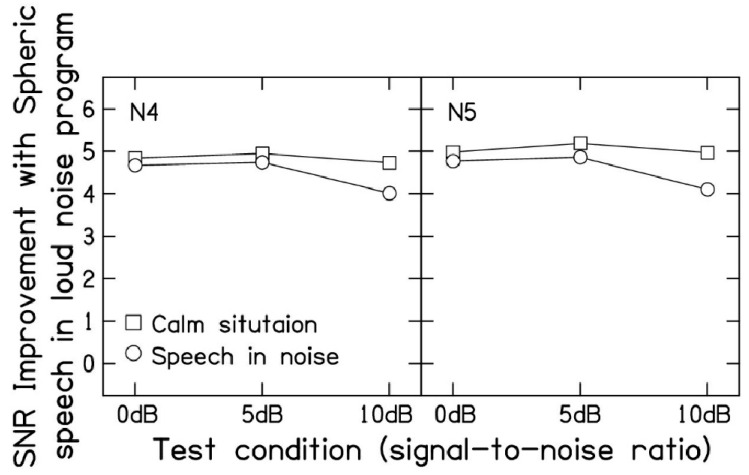
Signal-to-noise ration improvement (dB) with the spheric speech in loud noise program relative to calm situation (squares) and speech in noise programs (circles) for N4 (**left**) and N5 (**right**) audiograms across the 0, 5, and 10 dB SNRs.

Figure 3 demonstrates the SNR improvement provided by the spheric speech in loud noise program compared to the calm situation and speech in noise programs at the 0, 5, and 10 dB SNRs. The results indicate that the spheric speech in loud noise program consistently increases the SNR of the aided signal. On average, the spheric speech in loud noise program outperformed the calm situation program by 4.8 dB for the N4 audiogram and 5.0 dB for the N5 audiogram across SNR levels. Similarly, when compared to the speech in noise program, the spheric speech in loud noise program offered an average benefit of 4.5 dB for N4 and 4.6 dB for N5 audiograms across SNRs. It is worth noting that the advantage of spheric speech in loud noise over the speech in noise program was smaller in magnitude at the 10 dB SNR as compared to the 0 and 5 dB SNRs due to differences between the calm situation and speech in noise programs.

**Figure 4:**
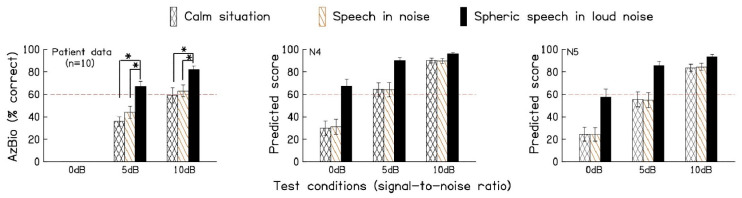
The left panel shows average sentence recognition scores for 10 hearing-impaired patients using three different hearing aid programs in the 5 and 10 dB signal-to-noise ratio (SNR) multi-talker babble conditions. The middle (N4) and right (N5) panels display the predicted speech scores, as calculated by HASPI v2, for the three programs in multi-talker babble conditions with SNRs of 0, 5, and 10 dB. Error bars represent the standard errors of the mean. * = *p* < 0.05.

Figure 4 shows average sentence recognition scores for the ten patients using three different hearing aid programs at the 5 and 10 dB SNRs. These results can be compared with the HASPI v2 model’s predicted scores that are illustrated in the other two panels for N4 and N5 audiograms. Note that patient scores were not measured at the 0 dB SNR. The results show that HASPI v2 predicted scores are approximately 10 to 15% points higher than those measured for the five bilateral and five unilateral hearing-impaired patients.

## 4. Discussion

This study evaluated speech recognition for hearing-impaired individuals, five with bilateral hearing loss and five with unilateral hearing loss, and a CI in the contralateral ear that was not used during speech intelligibility measurements. The hearing-impaired ear(s) were fitted with a Phonak Audéo Sphere Infinio 90 hearing aids, programmed with three different settings: the calm situation, speech in noise, and spheric speech in loud noise programs. Given that many participants scored below 60% with conventional listening programs, they would qualify for cochlear implantation. However, the spheric speech in loud noise program enabled most participants to achieve scores above 60% correct. These findings raise two crucial questions:

First, given the advancements in DNN-based noise reduction technology, how should patients be qualified for cochlear implantation? Cochlear implants offer significant benefits to many individuals with hearing loss. On average, they can improve single-word recognition to 58% and sentence recognition to 75% in the implanted ear [21]. However, current Medicare criteria limit access to these devices for many who could benefit. The requirement for moderate to profound bilateral hearing loss excludes individuals with asymmetric hearing loss or single-sided deafness who could greatly benefit from cochlear implantation. The 60% cut-off on sentence recognition tasks will exclude patients with better performance in one ear and poorer performance in the other. These individuals might benefit from implantation in the poorer-performing ear, but the current criteria prevent it. To address these limitations and better assess a patient’s potential for benefit, sentence recognition tests in challenging listening environments, such as those with multi-talker babble and low SNRs (10 and 5 dB), were used to assess cochlear implant candidacy. This approach more accurately reflects the real-world noise conditions that patients encounter daily. By qualifying patients based on their performance in these realistic listening scenarios, we can ensure that cochlear implants are provided to those who will derive the most significant benefit. With the advancement in DNN-based noise reduction, lower SNRs—such as 0 dB—may be required to identify hearing-impaired patients, particularly those with asymmetrical hearing loss with one exceptionally well-performing ear, in need of cochlear implantation. Even a 0 dB SNR stimulus presentation may not be sufficient to qualify some hearing-impaired listeners for cochlear implantation, as DNN-based noise reduction can provide an additional 5 dB of SNR improvement (see Figure 3). A limitation of this study is the lack of speech perception data at a 0 dB SNR. SNR analysis and HASPI modeling suggest that some patients might achieve scores below 60% at this level. Alternatively, CMS criteria could be revised to include ear-specific evaluations using single-word stimuli in quiet. This change would account for the potential of advanced hearing aid technologies, such as DNN-based noise reduction, to mitigate the need for cochlear implantation in certain cases. Some individuals may excel in sentence recognition due to top-down processing and cognitive strategies but struggle with single-word tests. Consequently, each ear should be evaluated separately using single word stimuli such as consonant–vowel–consonant stimuli [22]. For these individuals, cochlear implants can significantly improve access to sound, reducing cognitive load, and enhancing overall listening performance.

Second, given the significant improvement in speech perception achieved with DNN-based noise reduction, should cochlear implantation be reconsidered for individuals with audiograms and speech perception scores similar to our study participants? For instance, our cohort of 10 patients had a pure-tone average of 69 dB HL and an average speech perception score of 83% in quiet conditions when using the calm situation program. An analysis of SNRs indicates that the current DNN-based noise reduction technology offers a substantial 4–5 dB improvement over traditional speech in noise programs. This enhancement can be further optimized through the integration of directional microphones, especially in real-world settings where speech and noise sources are spatially distinct leading to SNR improvements of approximately 8.5 dB [14]. Thus, the advancement of noise reduction technologies in hearing aids has the potential to delay or even eliminate the need for cochlear implantation in some hearing-impaired individuals.

Eventually CIs can derive the benefit of DNN-based noise reduction technology by incorporating this technology in their speech processors. While most CI users perform well in quiet listening conditions [22], many struggle in noisy environments, even at relatively favorable SNRs of 10 or 5 dB. The combination of DNN-based noise reduction and directional microphones could significantly improve speech perception for CI users in challenging listening situations.

While the initial results comparing DNN-based noise reduction with traditional algorithms are encouraging, a few challenges remain for the widespread adoption of DNN-based technologies: DNNs are computationally intensive, which can significantly increase battery power consumption. This could limit the wear time of smaller hearing aids, such as receiver-in-canal or behind-the-ear devices. Increasing battery size is a potential solution, but it can lead to bulkier and less esthetically pleasing devices. A more efficient approach is to selectively engage DNN-based noise reduction only when necessary. This can be achieved through the automatic detection of noisy environments. For instance, the Phonak Audéo Sphere Infinio 90 utilizes the Autosense OS to monitor ambient noise levels and activate DNN-based noise reduction. This strategy optimizes battery life by limiting the use of computationally intensive DNNs to noisy situations, providing a balance between performance and power consumption. The effectiveness of DNN-based noise reduction may be compromised in open-fit hearing aid configurations [23]. The direct sound from the noisy environment mixes with the denoised signal from the hearing aid in the ear canal, potentially reducing the perceived benefit of the noise reduction technology. Ashkanichenarlogh et al. evaluated various signal processing methods used in modern hearing aids, comparing them to the DNN-based noise reduction algorithm [24]. The performance was assessed across different hearing loss categories (N2, N3, N4, and N5) and various venting conditions (open, vented, power, and custom shells with closed or 1 mm vent). The study concluded that DNN combined with beamforming outperformed traditional noise reduction. Improvements were observed across all venting conditions, but as expected, the benefits were reduced as venting increased. Although power domes were used for this study population, investigators anticipate that benefits would increase as venting decreases. Additionally, it can be inferred that a participant undergoing a cochlear implant candidacy evaluation would likely be fitted with near-occluded or fully occluded hearing aids, where the benefits of signal processing technologies like DNN-based noise reduction would be more pronounced.

To fully realize the potential of future DNN-based noise reduction improvements, it is crucial to address these challenges through advancements in hardware and software, as well as innovative hearing aid designs. One potential solution is the use of adaptive vent technology where the mechanical vent can be opened or closed based on the noise level around the hearing aid user. However, the market currently does not offer this technology with the ability to meet the audibility needs of listeners with severe-to-profound hearing loss.

Bench evaluation using SNR analysis [15] quantified the SNR improvement provided by the hearing aid and the DNN-based noise reduction algorithm. Slight differences in SNR benefits between N4 and N5 audiograms may be due to variations in gain and automatic gain compression. While SNR analysis provides valuable insights into noise reduction, it cannot predict speech intelligibility. The Hagerman–Olofsson method was designed to predict SNR for a mixture of speech and noise. While improvements in SNR can make speech easier to understand, this relationship is not necessarily direct. Noise reduction (NR) algorithms can inadvertently diminish speech quality by introducing distortion artifacts and reducing overall signal loudness, while simultaneously attenuating background noise. This can lead to no significant improvement, or even a deterioration, in speech intelligibility [10,25]. Therefore, to complement the speech intelligibility data gathered from participants, a model-based prediction of speech intelligibility, HASPI v2, was incorporated.

HASPI v2 is a sophisticated computational model that can be used to predict how well a hearing aid will perform in various listening environments [16]. By modeling the complex interactions between hearing loss, hearing aid processing, and various acoustic environments, HASPI v2 can be used to predict speech intelligibility. Our results show that the HASPI v2 predicted speech perception scores for the N4 and N5 audiograms, by a slightly greater magnitude than the actual speech perception scores measured for our patients. It is possible that individuals with overall poorer speech perception, relative to their peers with similar audiograms, are more likely to be referred for cochlear implantation because speech perception scores vary considerably among individuals with comparable audiograms. Given the impracticality of measuring speech perception for all types of audiograms and in all listening conditions and acoustic environments, tools such as SNR analysis and HASPI v2 provide valuable methods to predict the noise reduction capability of hearing aids and provide a rough estimate of speech perception for patients.

A major limitation of our study is the relatively small sample size and the predominance of moderate-to-profound, sloping sensorineural hearing loss among our participants. To further validate the benefits of DNN-based noise reduction programs and their potential impact on cochlear implant candidacy, future studies should include larger sample sizes encompassing a wider range of hearing loss severities.

## 5. Conclusions

Traditional noise reduction features in hearing aids have shown limited improvements in SNRs and speech perception due to potential signal distortions [8,9]. Patients with aided speech understanding below the threshold that defines cochlear implant candidacy are therefore referred for implantation. DNN-based noise reduction algorithms enable some patients to achieve speech recognition scores above the 60% threshold. A widespread adoption of DNN technology should encourage discussions on whether cochlear implant candidacy criteria should be revisited. Future research should explore the full potential of DNN-based noise reduction across larger sample sizes and by testing various levels of noise reduction and evaluating the maximum achievable improvement, especially for cochlear implant users who are highly susceptible to background noise interference.

## Figures and Tables

**Table 1:** PTA = pure tone average. Subject demographics for the ten hearing impaired patients included in this study.

		PTA _(0.5,1,2,4 kHz)_	
Subjects	Age (Years)/Gender	Left Ear	Right Ear
B1	61/Male	81.25	70
B2	78/Male	63.75	57.5
B3	75/Male	50	68.75
B4	62/Female	92.5	73.75
B5	68/Male	66.25	66.25
U6	34/Male	83.75	-
U7	66/Male	57.5	-
U8	69/Male	-	75
U9	63/Male	78.75	-
U10	78/Male	52.5	-

## Data Availability

The original contributions presented in this study are included in the article. Further inquiries can be directed to the corresponding author.
